# Development of an instrument to analyze organizational characteristics in multidisciplinary care pathways; the case of colorectal cancer

**DOI:** 10.1186/s13104-015-1084-1

**Published:** 2015-04-09

**Authors:** Dorine J Pluimers, Ellen J van Vliet, Anne GH Niezink, Martijn S van Mourik, Eric H Eddes, Michel W Wouters, Rob AEM Tollenaar, Wim H van Harten

**Affiliations:** Department Health Technology and Services Research, University of Twente, School of Management and Governance, Drienerlolaan 5, 7522 NB Enschede, the Netherlands; Sint Franciscus Hospital, Kleiweg 500, 3045 PM Rotterdam, the Netherlands; Deventer hospital, Nico Bolkesteinlaan 75, 7416 SE Deventer, the Netherlands; The Netherlands Cancer Institute – Antoni van Leeuwenhoek hospital, Plesmanlaan 121, 1066 CX Amsterdam, the Netherlands; Leiden University Medical Center, Albinusdreef 2, 2333 ZA Leiden, the Netherlands

## Abstract

**Background:**

To analyze the organization of multidisciplinary care pathways such as colorectal cancer care, an instrument was developed based on a recently published framework that was earlier used in analyzing (monodisciplinary) specialist cataract care from a lean perspective.

**Methods:**

The instrument was constructed using semi-structured interviews and direct observation of the colorectal care process based on a Rapid Plant Assessment. Six lean aspects that were earlier established that highly impact process design, were investigated: operational focus, autonomous work cell, physical lay-out of resources, multi-skilled team, pull planning and non-value adding activities. To test reliability, clarity and face validity of the instrument, a pilot study was performed in eight Dutch hospitals.

**Results:**

In the pilot it proved feasible to apply the instrument and generate the intended information. The instrument consisted of 83 quantitative and 24 qualitative items. Examples of results show differences in operational focus, number of patient visits needed for diagnosis, numbers of staff involved with treatment, the implementation of protocols and utilization of one-stop-shops. Identification of waste and non-value adding activities may need further attention. Based on feedback from involved clinicians the face validity was acceptable and the results provided useful feedback- and benchmark data. The instrument proved to be reliable and valid for broader implementation in Dutch health care. The limited number of cases made statistical analysis not possible and further validation studies may shed better light on variation.

**Conclusions:**

This paper demonstrates the use of an instrument to analyze organizational characteristics in colorectal cancer care from a lean perspective. Wider use might help to identify best organizational practices for colorectal surgery. In larger series the instrument might be used for in-depth research into the relation between organization and patient outcomes.

Although we found no reason to adapt the underlying framework, recommendations were made for further development to enable use in different tumor- and treatment modalities and in larger (international) samples that allow for more advanced statistical analysis. Waste from defective care or from wasted human potential will need further elaboration of the instrument.

**Electronic supplementary material:**

The online version of this article (doi:10.1186/s13104-015-1084-1) contains supplementary material, which is available to authorized users.

## Background

Increasingly multidisciplinarity is stressed to be an important characteristic in various pathways in hospital care. Efficiency is one of the aspects of quality identified by the Institute of Medicine and increasing financial pressure underlines the importance of this aspect. Comparing organizational aspects of multidisciplinary care pathways can be helpful in identifying best practice examples and areas for improvement. Operations management is the research field concerned with the delivery process of products and services. Under this heading, one of the approaches frequently adapted in healthcare is known as ‘lean thinking’. Spread beyond its Japanese roots the characteristics of this approach are to meet customer demand instantaneously, with perfect quality and no waste of resources. It results in items flowing rapidly and smoothly through the delivery process [1,2].

Analyzing differences and similarities in hospital organization can be the starting point for in-depth research on the possibilities to improve efficiency and patient related clinical outcomes [3].

Recently, van Vliet et al. [4] developed an analytic framework on the basis of six main aspects based on which treatment processes can be measured from a lean thinking perspective; this published framework was used in an adapted form as an evaluation instrument to analyze cataract care and involves six basic operational aspects of lean thinking that highly impacts process design:

1. *Operational focus* according to lean is to reduce the time line of the process by removing all non-value adding activities.
2. *Autonomous work cells* reduce the risk of interference from other processes by organizing all involved workstations as much as possible into one work cell.
3. The *physical lay-out of resources* aligns all activities in the sequence of the colorectal care process to prevent delays, caused by coordinating consecutive activities.
4. Team members are as *multi-skilled as possible*. A lean care team combines a maximum flexibility to conduct tasks interchangeably, as far as competence allows, with a minimal transfer of information.
5. *Pull planning* is used to couple resources to activities directly and on demand. Separate coordination of activities can lead to waiting times for patients or staff.
6. *Non-value adding activities* are eliminated as much as possible, such as over processing, over production, motion, transportation, waiting and inventory depository [4,5].

Cataract surgery, however, is a monodisciplinary and relatively low-complex care process, and it is relevant to ascertain whether this framework can also be used to draft an evaluation instrument for more complex, multidisciplinary care pathways.

Colorectal cancer is the fourth most common cancer in men and the third most common cancer in women worldwide [6]. Previous studies have reported rapid increases in colorectal cancer incidence rates, particularly in economically transitioning countries [7]. The demand for colorectal cancer care will therefore be higher in the next decades [8]. This calls for efficiently organized care requiring processes, people, skills and resources coordinated and integrated into one coherent service system. This coordination is at the core of what is called ‘operations management’ (OM) [2].

As colorectal cancer care (CRC) is a more complex care process than cataract care, it is necessary to test how the colorectal surgery process can be analyzed using the framework from van Vliet et al. [4]. The developed survey instrument for the complex care process of colorectal cancer care is based on all aspects of colorectal surgery and consists of a topic list for a semi-structured interview and an observation format. Required data from this topic list were subsequently restored as data in the framework of six basic operational aspects of lean thinking. In addition to this framework collected data can be used for a broader purpose, in terms of benchmarking improvement areas and provide insight into the CRC pathway. Regardless the potential feedback that could be provided, the purpose of this study was restricted to piloting the developed instrument for the analysis of the organization of colorectal cancer surgery. Reliability, clarity and face validity of the instrument was tested in eight hospitals in the Netherlands. First results of differences and similarities in the organization for CRC are presented.

## Methods

### Study design

To systematically collect data, we based the draft instrument on the recently developed framework of van Vliet et al. involving six basic operational aspects of lean thinking that highly impact process design, as described by Ohno, Womack and Jones and Liker: operational focus, autonomous work cell, physical lay-out of resources, multi-skilled team, pull planning and elimination of waste [9-11].

Based on these main aspects, a semi-structured interview was developed and specifically adapted to the organizational characteristics of the colorectal care process. For one of the six aspects, non-value adding activities, a verification of the Enhanced Recovery After Surgery (ERAS) was included, as this is an evidence-based example of value or its absence when low scoring, but could also be used as standardized work. [5].

**Figure 1:**
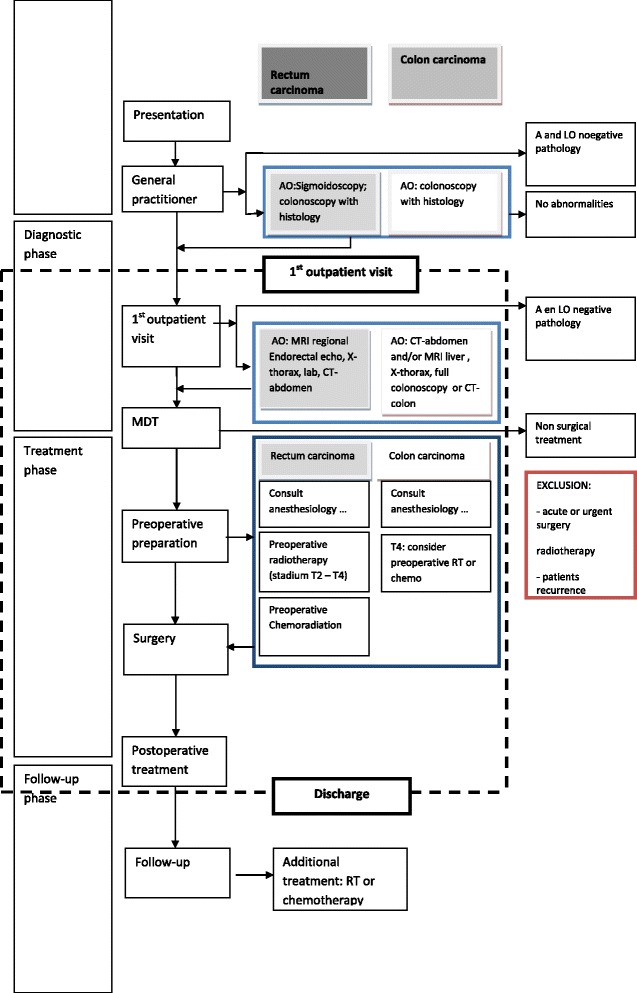
**Overview of the colorectal pathway in a flowchart.**

For a complete coverage of the colorectal process, the topic list had to be translated for the clinical setting at hand and was developed for influencing elements of the existing pathway: ‘general environmental context’ (32 questions), ‘resources’ (10 questions), ‘multidisciplinary team (MDT) (8 questions), ‘surgery’ (5 questions), ‘nursing ward’ (7 questions) and ‘fast track program’ (6 questions). Data collection took place at all involved departments, the outpatient clinics for surgery and gastroenterology, the operating theatre, radiology and nursing wards. The process stage studied was delineated from the first diagnostic outpatient consultation until the day of discharge from the hospital after surgery: the diagnostic phase with staging of the disease, the preoperative phase to prepare the patient for surgery and the inpatient phase, including the admission and the peri- and postoperative care activities (see Figure 1).

In addition to the interview, we integrated an observation format based on the Rapid Plant Assessment (RPA, adapted version), developed by Goodson et al. [12]. The tool originates from manufacturing and aims to assess organizations to rate a plant on lean attributes with an educated team. RPA items were listed on an observation format primarily aiming to generate more detailed information on the framework aspects of operational focus and physical lay-out. The adapted items of the RPA in the observation format comprised the topics ‘customer satisfaction’, ‘safety environment, cleanliness and order’, ‘visual management system’, ‘logistics, capacity, planning and scheduling, ‘use of space, movement of materials, ‘levels of inventory and work in process’, ‘teamwork and motivation’, ‘condition and maintenance of equipment and tools’, ‘management of complexity and variability’ and ‘product line flow’. Each topic was scored on a five point Likert scale classed in ‘not’, ‘limited’, ‘average’, ‘often’ and ‘always’.

An expert panel of three surgeons (EE, MW, RT), specialized in colorectal surgery and involved in the Dutch Surgical Colorectal Audit (DSCA), two researchers with expertise on operations management and health sciences (DP, WH), and a research physician with expertise on the oncology treatment (AN) verified the topic list for the semi-structured interview and the observation format.

To test feasibility, the surgeons were asked to consider the extent to which the questions and components could be unambiguous understood and applied; they also evaluated the extent to which the questions adequately represented the domain of colorectal cancer surgery.

To judge inter-rater reliability, two researchers (DP, AN) independently executed the interviews and observations in one categorical, one general, and one university hospital in the Netherlands and compared the outcomes afterwards. Differences in the execution of the interview and observation formats were compared and adjusted in consensus.

Subsequently, a pilot study was performed in eight Dutch hospitals with the purpose to test the reliability and face validity of the interview and observation format and if possible generate improvement suggestions. The invitation to participate was directed at the GI surgeon, the manager of the department was asked for data collection permission and various medical and nursing staff was interviewed. A hospital site visit to collect the data took three to five days.

**Figure 2:**
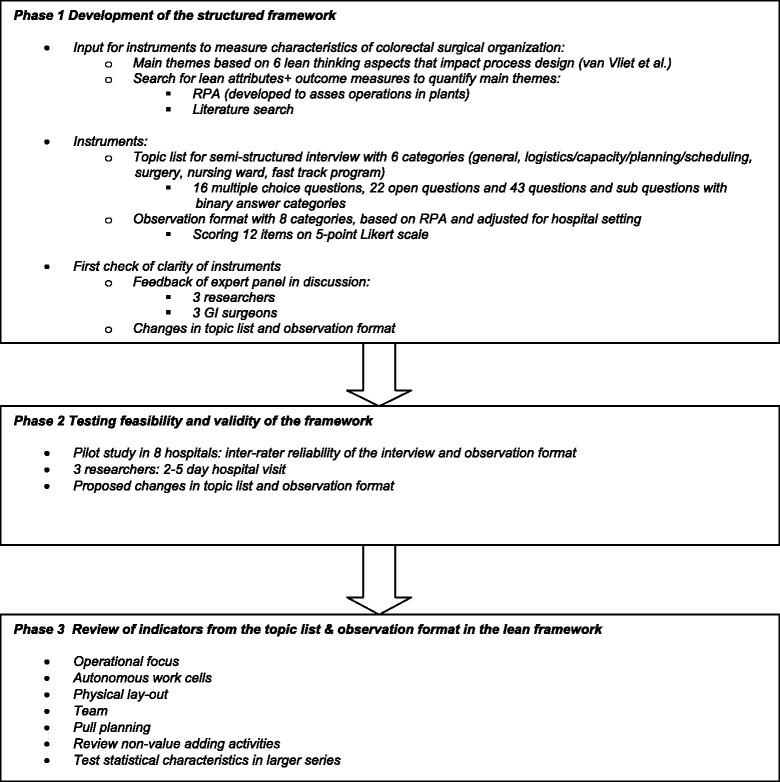
**Overview of study design.**

Four researchers (WH, AN, MM, DP) selected the outcome examples for the six headings of the framework in consensus with the same surgeons and researchers who participated in the panel. The study design is displayed in Figure 2 and the evaluation instrument is provided in Additional file 1.

### Data collection process and synthesis of results

Raw interview and survey data were collected in a semi-structured format and subsequently tabulated on a data extraction sheet (SPSS 19.0). A descriptive statistics analysis was performed for a first impression on validity.

### Approval

The research was performed according to the Dutch ethical review guidelines in medical research; ethical approval is not needed for process analysis or anonymous patient data. The Dutch Institute for Clinical Auditing (DICA) authorized the use of the anonymised data for the current research.

## Results

The data collection was executed according to plan. The accessibility of data and relevant persons in the hospitals and the departments was adequate. The semi-structured interviews and the observations were performed within the scheduled time. The interview took one to one and a half hours to complete per interviewee. The observations at the outpatient clinics for surgery and gastroenterology, operating theatre, radiology and nursing ward, took, depending on the flexibility in which arrangements on access could been made, two to five days to complete.

### Feasibility: adjustments in the interview and observation format

From the panel of three GI surgeons and three researchers, recruited to review the semi-structured interview and observation format, three did not recommend changes to the semi-structured questionnaire and observation formats.

Three panel experts (EE, MW, WH) suggested refinements such as changing the open questions into multiple choice or semi-open, in order to increase the efficiency of the interview and the comparability of the organizational characteristics. Questions were added to differentiate for hospital type, education level, bed capacity and offered treatments concerning CRC. Based on the pilot the definition of multidisciplinary consultation was refined. The colorectal care pathway was narrowed down by including only questions concerning elective care.

### Inter-rater reliability

Two researchers (DP, AN) utilized the questionnaire and observation format to score a sample of the participating hospitals independently and compared their results afterwards. The two researchers interviewed the same persons in each hospital. An agreement of 75% on level of response was reached for the 67 multiple choice questions and observations (60 questions). When a discrepancy occurred it was discussed with the expert panel until consensus was reached and the question was reformulated. The survey instrument is presented in Additional file 1.

### Results of the pilot categorized per main aspect

The instrument consisted of 83 quantitative, 24 qualitative items and a rapid plant assessment was conducted during observations on site. Examples of differences and similarities in the CRC organization are presented following the six main aspects.

#### Operational focus

In a lean incorporated hospital or pathway the focus is on medical performance (quality), reduction of lead times (flow) and reduction of costs (economic efficiency) at the same time. Examples of responses in operational focus for the CRC were whether the hospital focused its strategy on medical quality, operational aspects or both (items 9 and 10). Further, in order to obtain an impression on operational focus, the number of patient visits from the first hospital visit until discharge after surgery were scored following registrations in the Electronic Health Record (EHR). Hospital visits were defined as the number of patient visits to a physician and for diagnostic tests (including colonoscopy and MRI/PET and excluding visits for laboratory tests). In order to provide a complete picture of the utilization of the system’s assets, the number of patient visits should be combined with other indicators from the instrument, such as number of days from first contact to discharge, length of stay and bed occupancy rates.

The results on operational focus and mean number of patient visits needed for diagnosis, for 8 participating hospitals are presented in Table 1. Hospital 1, 6, 7 and 8 answered to focus on medical as well as operational content. Hospital 2, 4 and 5 declared to focus mainly on medical quality, in contrast to hospital 3 which declared a mainly operational focus. For the mean number of patient visits per episode, both the highest (7.6, n = 40) and lowest (5.3, n = 30) scores were found in hospitals indicating to give priority to medical quality.

**Table 1:** **Examples of differences and similarities in eight Dutch hospitals on colorectal organization**

**Hospital number**	**Flowchart for rectum**	**Flowchart for colon**	**Operational focus on medical content, operational content or both**	**Operational focus: mean number of patient visits (mean overall: 6,72)**	**AWC: Multidisciplinary outpatient clinic**	**AWC: use of dedicated sessions**	**Physical layout: safety, cleanness and order (RPA)**	**Physical layout: visual management system (RPA)**	**Team: number of staff involved with diagnosis**	**Pull: one stop shop for diagnosis**	**Non-value adding activities**
**1**	yes	Yes	both	7.5	no	yes	3	5	4	no	yes, prbERAS
**2**	no	Yes	medical	5.6	no	no	4	3	4	no	yes
**3**	yes	Yes	operational	6.7	no	yes	3	2	2	yes	yes, prbERAS
**4**	yes	Yes	medical	5.2	no	yes	4	3	8	yes	yes
**5**	yes	Yes	medical	7.6	no	yes	3	3	3	no	yes
**6**	yes	No	both	6.	yes	no	4	3	4	no	yes, prbERAS
**7**	no	No	both	5.9	no	no	4	4	5	no	no
**8**	yes	Yes	both	mv	no	no	4	2	9	yes	yes

mv = missing value. AWC = Autonomous Work Cells, prbERAS = protocol based on ERAS.RPA = Rapid Plant Assessment.Rating RPA colums physical layout: 1 = not, 2 = low, 3 = average, 4 = often, 5 = always.

#### Autonomous work cells

Examples of questions regarding the work cell facility were whether the hospital utilized a pre scheduled multidisciplinary outpatient session for colorectal patients, how these were organized per week, the number of staff members involved with diagnosis and treatment and whether all diagnostics resources were available in the hospital (items 16–19, 33–40 and observation item 8).

Only one hospital (hospital 6) used such a multidisciplinary outpatient clinic. Fixed but separated session slots were used in four hospitals (hospital 1, 3, 4 and 5). The number of staff involved for diagnosis was the highest in hospital 8 (9 staff members) and the lowest in hospital 3 (2 staff members). These results are displayed in Table 1.

#### Physical lay-out

Differences and similarities in physical lay-out were explored and measured with the (RPA) observation score, focusing on safety environment, cleanness and order, the use of visual management in the CRC pathway and the use of space usage, transport and streamlining (observation items 5–7 and 9). The observations showed that most hospitals scored ‘average’ in the range ‘not/low/average/often/always’, with the exception of 2 hospitals who scored ‘often’ for safety environment, cleanness and order.

#### (Multi-skilled)Team formation

Differences and similarities were assessed with the questions into the organization of multidisciplinary team meetings, the number and professions of team members involved, the formation of the surgery team and the nursing team (items 20, 42–49, 50–52, 58, 59 and observation item 10). The number of team members involved with treatment ranges from 2 in hospital 3 to 9 in hospital 8 (Table 1).

#### Pull planning

Pull planning was assessed by the availability of one-stop shop for the diagnostic phase, how surgery was planned, whether clinical pathways were implemented, the presence of and adherence to flow charts and how complexity and urgencies were managed (items 4–6, 21 and observation items 11 and 12).

Observations were performed for in-depth information on management of complexity and urgencies, supply chain integration, and scheduling system of the outpatient clinic.

An example of a response on one-stop shop for diagnosis, is presented in Table 1. Hospitals 3, 4 and 8 are using one- stop shop for diagnosis, shaped by offering all diagnosis in one day.

#### Non-value adding activity

The implementation of Enhanced Recovery After Surgery (ERAS) was scored with no, yes or yes, an ERAS-based protocol (items 61–63). In Table 1 is shown, that some hospitals use the original ERAS protocol (hospital 2,4,5 and 8), some a protocol based on ERAS (hospital 1,3 and 6) and one is not using ERAS nor any alike (hospital 7).

In the interview suggestions were given to further reduce waste, such as considering the use of a dedicated endoscopist [13], the use of electronic health records, having a single patient file as it will reduce complexity and will enhance the smoothness of information flow [14] and start open access colonoscopy [15].

### Qualitative additions to the results

In addition to the quantitative results, additional notes were listed for RPA items in order to complement the total picture of the colorectal organization. For example, we show additional RPA notes for the topics ‘patient satisfaction’, ‘safety’ and ‘visual management system’.

#### Patient satisfaction

Information about patient satisfaction on CRC was traceable in three cases; on the website (1 hospital) and via a special heading in the website ‘your opinion’ (1 hospital), for one hospital was noted that it received a ‘3 stars’ degree for hospitality. One hospital indicated to have received awards for patient satisfaction though no information was found on the website.

#### Safety

One of the collected notes concerned the specific supply system of the operating theatre with larger (and often required) supplies was situated at a distance and smaller supplies nearby. This carried the risk that delays may arise and safety might be influenced. Another observed occurrence influencing waiting times and safety was observing devices lying around in the wards and operating rooms, whereby patients as well as employees make mistakes, or delays arise due to the cleaning up or search for specific devices.

#### Visual management system

Visual management was used, but still in early stages. For example, the ability to inform patients and staff about waiting times was only seen in few hospitals. Color signals were sometimes observed for medication processes and office availability.

Workinstructions and clinical guidelines were present, but observed in many ways. In one case as instruction files of many pages stored in (outlying) cabinets and in the other as visual (photo) presentations on the performance at the location of the operation.

Back office productivity charts were hardly found. When information was available, it was handwritten in most cases. As an exception, the operating theatre was often decorated with an (electronic) white board to visualize the schedule per operating room.

### An illustrative sample report to show how a hospital can be ‘scored’

To illustrate the potential of the instrument, in terms of identifying and benchmark improvement areas, a sample report is shown in Additional file 2. The first tables show demographic data, like hospital size and hospital type. Examples of indicators regarding efficiency and patient related outcome are presented comparing the hospital with the mean scores of other hospitals. The indicators in the sample report are only a small fraction of outcome examples that can be value for different stakeholders.

## Discussion and conclusions

This paper has demonstrated that it was possible to develop an instrument to analyze organizational and operational characteristics from a lean thinking perspective, for multidisciplinary complex pathways like oncological colorectal surgery. The survey took two to five days for site visit activities and in general all data were available within a reasonable period. In the pilot we tested inter-rater reliability and validity. Results demonstrate that the instrument provides sufficient detail and is capable to show differences in organization of colorectal surgery in the tested Dutch hospitals and the efforts are valuable for different stakeholders. Examples are whether or not to use a multidisciplinary outpatient clinic and one-stop-shop for diagnosis, the implementation state of flow charts and the number of patient hospital visits. By performing observations based on the RPA approach, qualitative information is added, complementing the data into a holistic “operations snapshot” of a hospital service.

The pilot in eight hospitals assisted us in visualizing the organizational differences and we were able to produce a first benchmark report as feedback; this can be further elaborated in future.

The instrument produces a rich database of a range of organizational characteristics (see Additional file 1) and provides a solid basis for further research in follow-up studies in larger series, and possibly international comparison. Research into the correlation between organizational characteristics and patient related outcomes could be a next stage in research [16].

Limitations of this study however should be considered. The initial framework was based on a cataract process, which is a process with rather low complexity. The oncological colorectal disease is characterized by multiple options in tumor type and staging. The therapy can consist of multiple treatments, for example curative or non-curative. The treatment of colon cancer and rectal cancer are also different and based on separate clinical guidelines resulting in different care processes [17]. Furthermore radiotherapy department was not yet included in this research. This would be especially interesting for the rectal cancer care pathway. It is therefore recommended that the instrument will be further adjusted for each cancer type and towards treatment modalities in future.

Safety has become a very important factor in healthcare and although also appearing in papers on lean management, it is also a discipline in itself. Although the topic safety was partly covered in the present study, it was limited to indicators regarding the developed framework.

Some topics might occur in several items of the framework. The issue of non-value adding activities appears in two of the six items of the framework: in its own section as well as in the one on operational focus, defined as the streamlining of the process through elimination on non-value adding activities. This indicates that further research into the use and attribution of indicators in the framework. Moreover, regarding non-value adding activities, an important aspect to consider is wasted human potential as lean also stands for engaging, encouraging and supporting frontline staff in continuously improving the way that work is done. Workforce engagement and participation in continuous improvement initiatives is an organizational characteristic that could help distinguish best practices and better understand the performance of different multidisciplinary pathways. Lean thinking is defined as meeting customer demand with perfect quality and implies care that is free of defects, such as hospital acquired infections, surgical complications and medication errors. These topics are partially addressed in the present study partly due to a lack of data. Although the topic is referred to in the framework (multiskilled team members), we advise more attention for these in future research. The identification and reduction of non-value adding activities, several approaches can be applied. One of these is the implementation of a fast-track protocol to structure processes. There is a large amount of scientific evidence that these protocols really enhance the recovery of the patient but data on its contribution to efficiency are scarce . Although in the current instrument questions are limited to binary a yes or no answers, it may be useful to explore the potential for quantitative scoring on the compliance to such protocols. Although elements from the RPA are added in the current study and actually prove to provide observational information, research into the value of the full RPA as a freestanding instrument and scoring/rating “leanness” of a healthcare organization, might also be interesting. In industry a concise RPA index-score can be calculated; so far we did not find evidence that the latter is feasible in health care. The use of the RPA in the present study was meant to generate additional, observational information [17]. Furthermore, a 5-point Likert scale was used to scale the RPA observations. Critical is whether the distance between each category is comparable. In terms of good research practice, an equidistant presentation is important, otherwise a bias in the analysis may result. For example, a five-point Likert scale as used in the current research with categories “Not”, “Limited”, “Average”, “Often”, and “Always” needs a separate analyses into the interpretation of the categories by respondents. Hence, the usefulness of the Likert scale should be elaborated in further research.

The number of hospital visits needed for diagnosis are presented as a mean. For a more meaningful comparison of hospitals, an indicator of variability should also be included, as the mean alone could be misleading and is susceptible to extreme values. A small sample size due to ethical review regulations was the reason that only a mean was presented and further research with larger sample size is recommended. Remaining, questions concerning costs could not be answered; possibly due to the introduction of price competition in the health insurance system in the Netherlands hospital management was reluctant to provide detailed financial information. However, further research into financial information and relations with organization differences could be of great value.

In this paper we used an instrument based on a lean framework in order to obtain insight in the organizational characteristics of multidisciplinary colorectal cancer care. Further validation in larger series will create opportunities to test correlations between organizational characteristics and patient related outcomes aiming to recommend best practices for colorectal surgery.

## Additional files

Additional file 1:
**Survey instrument (semi-structured interview).**
Additional file 2:
**Sample report.**
