# Supplementary material for: Development of an instrument to analyze organizational characteristics in multidisciplinary care pathways; the case of colorectal cancer
Source: BMC Res Notes. 2015 Apr 9;8:134. doi: 10.1186/s13104-015-1084-1 (PMC4396572; doi:10.1186/s13104-015-1084-1)
Supplement: Additional file 1: — Survey instrument (semi-structured interview). [file 13104_2015_1084_MOESM1_ESM.doc]

**INSTRUMENT: TOPIC LIST SEMI STRUCTURED INTERVIEW & OBSERVATION FORMAT**

**General characteristics:**

Name organization

Department

Place

Contact person

Phone number

Data collection period

Interview date

1. How is the colorectal cancer care organized in your hospital? (open question)
2. Is your hospital working with a multidisciplinary protocol or clinical pathway for colorectal cancer care? Yes/no
3. Is there a document available describing the clinical pathway? Yes/no

(If Yes, attach a document)

4. Is the hospital using a fast track protocol (meaning a protocol that is meant to promote an accelerated recovery after surgery) for example the ERAS protocol? Yes/no

5. Is this protocol available: Yes/no

(If Yes, attach a document)

6. To what extent is this protocol implemented in the hospital?

A. there are no protocols available

B. the written protocols are present

C. the written protocols are present and are showed in the patient record

D. the protocols are present and tracked by a checklist/database

E. the protocols are present, checked and feedback is given

F. Otherwise, namely ... ... ... ... ... ... ... ... ... ... ... ... ... ... ... ... ... ... ... ... ... ... ... ... ... ... ... ... ... ... ... ... ... ... ... ... ... ... ... ... ... ... ... ... ...

7. How is the surgical staff unit organized within the hospital? Paid employment, individual membership or staff membership?

A. Paid Employed

B. Staff membership

C. Individual membership

8. Is your hospital a training hospital for surgery? (multiple answers possible)

A. Yes, training hospital for medical students

B. Yes, training hospital for surgical interns

C. Yes, training hospital for fellows in surgical oncology

D. Yes, training hospital for fellows in GI surgery

9. Have there been important changes in 2010 in the organization of care around the oncological patient with a colorectal condition in your hospital? Yes/no

9a. If Yes, what were the changes? ………………………………………………………………………………………………………………………………………………………………………………………………………………………………………………………………………………………………………………………… 9b. If Yes, how have these influenced the process? ………………………………………………………………………………………………………………………………………………………………………………………………………………………………………………………………………………………………………………………… 10. Please score the influencing factors on choices around the organization of the care process; add factors if possible. Put behind the factor the sequence of importance, where number 1 represents the factor with the most influence on the process.

Patient satisfaction ... ... ... ... ...

Employee satisfaction ... ... ... ... ...

Capacity utilization ... ... ... ... ...

Cost control ... ... ... ... ...

Efficiency ... ... ... ... ....

Care quality ...

11. Could you please indicate how the first influencing factor has affected the care process? ………………………………………………………………………………………………………………………………………………………………………………………………………………………………………………………………………………………………………………………….

12. Does the supplied flowchart provide a good reflection of the colorectal care process in your hospital? Circle the answer: Yes/No

12a. Remarks: ……………………………………………………………………………………………………………………………………………………………………………………………………………………………………………………………………………………………………………………………………………………………………………………………………………………………………………………………………………………………………………………………………………………………………………………………………………………………………………………

13. Are these kind of flow charts used in your organization? Circle the answer: Yes/No

13a. If Yes, add a copy of the flow chart you use.

*(if this question is answered by the surgeon with NO, this question is also posed to the care manager)*

14. Is there a designated contact person per patient?

14a. If Yes, who fulfills this role?

Medical oncologist

Surgeon doctor

Specialized Nurse

Nurse practitioner

Physician assistant

Dedicated Case manager

Different namely, ... ... ... ... ... ... ... ... ... ... ... ... ... ... ... ... ...

14b. For which part(s) of the treatment process, the patient may turn to this (these) person(s)? ………………………………………………………………………………………………………………………………………………………………………………………………………………………………………………………………………………………………………………………… …………………………………………………………………………………………………………………………………………………………………………………………………………………………………………………………………………………………………………………………

15. Can you give an estimate of the proportion of patients treated in your hospital that are referred from a hospital elsewhere.…..%

16. Does the hospital use a multidisciplinary outpatient clinic for patients with oncological colorectal condition (where at least a Gastroenterologist, Surgeon and Radiotherapist are present)? Yes/No

17. Are there dedicated days or times on which these outpatient clinics are planned ? Yes/no

18. How many are scheduled for Oncology office outpatient clinics weekly? ... ... ... ... ... ... ... per week

19. How many days or half days of the Oncology outpatient clinics are reserved for patients with colorectal condition? …………

20. Which specialists are involved during the diagnostic phase (initial consultation after presentation to date tumor positive PA outcome)?

Gastroenterologist

GI Physician

Surgeon

Medical oncologist

Radiologist

Pathologist

Radiotherapist

Anesthesiologist

Nurse practitioner

Physician assistant

Stoma nurse

Case manager

Wound nurse

Other namely, ...

21. How are the diagnostics organized? ……………………………………………………………………………………………………………………………………………………………………………………………………………………………………………………………………………………………………………………….. …………………………………………………………………………………………………………………………………………………………………………………………………………………………………………………………………………………………………………………………

22. At what moment is the surgery scheduled?

At the moment the patient has had the colonoscopy

On the day of the final pathology diagnosis

On the day of the multidisciplinary team conference

On a day after the multidisciplinary team conference

Other namely, ... ... ... ... ... ... ... ... ... ... ... ... ... ... ... ... ... ... ... ... ... ... ... ... ... ... ... ... ... ... ... ... ... ... ... ... ... ... ... ... ... ... ... ... ... ... ... ... ... ... ... ... ... ... ... ... ... ... ... ... ... ... ... ... ... ... ... ... ... ... ... ... ... ... ... ... ... ... ... ... ... ... ... ... ... ... ... ... ... ... ... ... ... ... ... ... ... ... ... ... ... ... ... ... ... ... ... ... ... ... ... ... ... ... ... ... ... ... ... ... ... ... ... ... ... ... ... ... ... ... ... ... ... ... ... ... ... ... ... ... ... ... ... ... ... ... ... ... ... ... ... ... ... ... ... ... ... ... ... ... ... ...

22a. Concerning the total number of patients the surgery can actually be scheduled on the day, as indicated in the previous question? In ..... (percentage) of the total number of patients operated in 2009 in connection with a colorectal neoplasm.

23. Are there dedicated operating room slots for oncological colorectal surgery ? Yes/no

23a. If Yes: how many half days are weekly scheduled for colorectal surgery? ... ... ... ... ... ... ... ...

23b. If no: Are places reserved/scheduled for colorectal surgery? Yes/no

24. Are all patients with colorectal carcinoma operated by specialized surgeons?

Yes, all patients are operated under the supervision of a specialized surgeon

Yes, except in the emergency cases

No

25. Are all elective patients with Colorectal carcinoma operated by specialized surgeons? Yes/no

26. How much time per patient is reserved for the total of the following activities: preparation, OK and changeover times?

26a. Hemicolectomie ... ... ... ... ... ... ... ... ... ... ... ... minutes

26b. Sigmoid resection ... ... ... ... ... ... ... ... ... ... ... ... minutes

26c. Low anterior resection ... ... ... ... ... ... ... ... ... ... ... ... minutes

26d. APR …………………………….. minutes

26e. Laparoscopic surgery ... ... ... ... ... ... ... ... ... ... ... ... extra minutes

27. Can you estimate the number of days between the first (telephone) contact with the hospital and the first outpatient clinic visit? Yes/No

27a. Minimal. ... ... ... ... days

27b. Average ... ... ... ... days

27c. Maximum ... ... ... .... days

27d. What determines the number of days or explains why it can take longer or shorter than expected? ……………………………………………………………………………………………………………………………………………………………………………………………………………………………………………………………………………………………………………………………………………………………………………………………………………………………………………………………………………………… 28. Can you estimate the number of days between the first visit outpatient clinic after referral and colonoscopy? Yes/No

28a. Minimal. ... ... ... ... days

28b. Average ... ... ... ... days

28c. Maximum ... ... ... .... days

28d. What determines the number of days or explains why it can take longer or shorter than expected? …………………………………………………………………………………………………………………………………………………………………………………………………………………………………………………………………………………………………………………………………………………………………………………………………………………………………………… ...

29. Can you estimate the number of days between the colonoscopy and the final pathology based diagnosis (PA outcome)? Yes/No

29a. Minimal. ... ... ... ... days

29b.. Average ... ... ... ... days

29c. Maximum ... ... ... .... days.

What determines the number of days or explains why it can take longer or shorter than expected? ……………………………………………………………………………………………………………………………………………………………………………………………………………………………………………………………………………………………………………………………………………………………………………………………………………………………………………………………………………………… 30. Can you estimate the number of days between the pathology result and the case discussion in the pre-operative multidisciplinary conference? Yes/No

30a. Minimal. ... ... ... ... days 30b. Average ... ... ... ... days 30c. Maximum ... ... ... .... days 30d. What determines the number of days or explains why does it can take longer or shorter than expected? ……………………………………………………………………………………………………………………………………………………………………………………………………………………………………………………………………………………………………………………………………………………………………………………………………………………………………………………………………………………… 31. Can you give an estimate of the number of days between the treatment decision and the operation date? Yes/No 31a. Minimal. ... ... ... ... days 31b. Average ... ... ... ... 31c. days. Maximum ... ... ... .... 31d days. What determines the number of days or explains why it can take longer or shorter than expected? ……………………………………………………………………………………………………………………………………………………………………………………………………………………………………………………………………………………………………………………………………………………………………………………………………………………………………………………………………………………… 32. Can you give an estimate of the number of days between the operation date and dismissal? Yes/No 32a. Minimal. ... ... ... ... days 32b. Average ... ... ... ... days 32c. Maximum ... ... ... .... days 32d. What determines the number of days or explains why it can take longer or shorter than expected? ………………………………………………………………………………………………………………………………………………………………………………………………………………………………………………………………………………………………………………………………………………………………………………………………………………………………………………………………………………………

**Logistics, Capacity, Planning and Scheduling.**

33. Outpatient consultation GE: there are places/slots reserved for oncological colorectal patients: Yes/No if yes, how many places are reserved per week? ……………………………………………

34. Outpatient consultation surgery: there are slots reserved for oncological colorectal patients: Yes/No if yes, how many places are reserved per week? …………………………………………

35. Radiology, X-thorax there are slots reserved for oncological colorectal patients: Yes/No if yes, how many places are reserved per week? ………………………..

36. Radiology, CT-abdomen there are slots reserved for oncological colorectal patients: Yes/No if yes, how many slots are reserved per week? ……………………………………………

37. Radiology, MRI there are places reserved for oncological colorectal patients: Yes/No if yes, how many places are reserved per week? ……………………………………………

38. Radiology, endo-echo there are slots reserved for oncological colorectal patients: Yes/No if yes, how many slots are reserved per week? ……………………………………………

39. Consulting Radio therapist

39a. There are slots reserved for oncological colorectal patients: Yes/No

39b. If Yes, how many slots are reserved per week? ……………………………………………

40. What time in minutes per patient (pp) is reserved for the following activities:

40a. First Outpatient visit: ... ... ... ... ... ... ... ... ... ... ... minutes ; by which specialty is the patient seen: ... ... ... ...

40b. Outpatient visit communicating the diagnosis: ... ... ... ... ... ... ... ... ... ... ... minutes.

By which specialty is the patient seen at that time: ... ... ... ... ... ... ... ... ... ... ... ... ...

40 c. Follow–up visit ... ... ... ... ... ... ... ... ... ... ... minutes... ... ... ...outpatient consultations/pp By which specialty communicating the diagnosis is the patient at that time seen: ... ... ... ... ... ... ... ... ... ... ... ... ... ....

40d. Coloscopy: (GE) ... ... ... ... ... ... ... ... ... ... ... minutes

40e. Echo: (RAD) ... ... ... ... ... ... ... ... ... ... ... minutes

40f. MRI: (RAD) ... ... ... ... ... ... ... ... ... ... ... minutes

40g. CT: (RAD) ... ... ... ... ... ... ... ... ... ... ... minutes

40h. X-Thorax: (RAD) ... ... ... ... ... ... ... ... ... ... ... minutes

41. How often does a patient stop by before a treatment decision is agreed?

41a. Minimal. ... ... ... ... time(s), + reason: ... ... ... ... ... ... ... ... ... ... ... ... ... ... ... ... ... ... ... ... ... ... ... ... ... ... ... ... ... ... ... ... ... ... ... ...

41b. Average ... ... ... ... time(s), + reason: ... ... ... ... ... ... ... ... ... ... ... ... ... ... ... ... ... ... ... ... ... ... ... ... ... ... ... ... ... ... ... ... ... ... ... ...

41c. Maximum ... ... ... .... time(s), + reason: ... ... ... ... ... ... ... ... ... ... ... ... ... ... ... ... ... ... ... ... ... ... ... ... ... ... ... ... ... ... ... ... ... ... ... ... Multi Disciplinary Team conference (MTC)

42. How often is a MDT (pre/post surgically operatively) planned in the process for patients with COLON Carcinoma (from initial consultation until dismissal MDL outpatient clinic)? ………………. Time

43. How often is a MDT planned in the process for patients with RECTUM Carcinoma (from initial consultation to dismissal)? ………………. Time

44. How are the MDT’s organized? GE specific / according to the national guideline?

45. Who are involved? Gastroenterologist/ Medicine MDL-doctor Surgeon Medical oncologist, Radiologist, Pathologist, Radiotherapist Nuclear, Gyneacologist Specialized Nurse a ... ... ... ... ... ... ... ... ... ... ... ... ... ... ... ... ... ... ... ... ... ... ... ... ... ... Otherwise,...

46. Are all patients with colon carcinoma preoperatively discussed? Yes/No

47. All patients with rectum carcinoma discussed preoperative Yes/No

48. How are patients selected for the MDT and are there in-and exclusion criteria? ……………………………………………………………………………………………………………………………………………………………………………………………………………………………………………………………………………………………………………………………………………………………………………………………………………………………………………………………………………………… 48a. If Yes, what are the criteria? ………………………………………………………………………………………………………………………………………………………………………………………………………………………………………………………………………………………………………………………………………………………………………………………………………………………………………………………………………………………

49. Is the conclusion of the MDT registered? Yes/No

49a. If Yes: Is this conclusion digitally available? Yes/No

**SURGERY**

50. Is there a dedicated surgical team, including an anesthesiologist? Yes/No

50a. What size has this OK team and what officers are involved?

1. ………………………………………………………………………………………….

2. ………………………………………………………………………………………….

3. ………………………………………………………………………………………….

4. ………………………………………………………………………………………….

5. ………………………………………………………………………………………….

6. ………………………………………………………………………………………….

51. Are there dedicated surgical teams for this type of surgery? Yes/No

52. Is there dedicated staff for laparoscopic surgeries? Yes/No

53. Is there a standard training for this staff? Yes/No

53a. If Yes, what training? ………………………………………………………………………………………………………………

54. Are there operating rooms specially equipped for laparoscopic surgeries? Yes/No

**NURSING DEPARTMENT (WARD)**

55. Is a specialized nurse available in the hospital for the GE/Colorectal carcinoma? Yes/ No

56. Is there a specific GE Nursing Department?

Yes, only surgical GE

Yes, GE/surgical Department

No, different types of patients per nursing ward

57. There are outpatient wound clinics? Yes/No

58. There are dedicated nurses? Yes/No

59. What is the ratio of (registered) nurses per bed at the wards where colorectal patients are treated?

59a. Day shift: ... ... ... ... ... ... ... ... ... ... ... ... ... ... ... ... ... ... ... ... ... ... ... ... ... ...

59b. Evening shift: ... ... ... ... ... ... ... ... ... ... ... ... ... ... ... ... ... ... ... ... ... ... ... ... ...

59c. Night shift: ... ... ... ... ... ... ... ... ... ... ... ... ... ... ... ... ... ... ... ... ... ... ... ... ....

60. What are the costs of one hospital in-patient day? € ……………………………………………………

**FAST RECOVERY PROGRAM**

61. Does your hospital work with a fast track program/fast recovery program based on the ERAS protocol or making use of the ERAS protocol?

Yes,

Yes, adapted protocol based on the ERAS protocol

Yes, different from ERAS, namely ... ... ... ... ... ... ... ... ... ... ... ... ... ... ... ... ... ... ... ... ... ... ... ... ... ... ... ... ... ... ... ... ... ... ... ... ... ... ... ... ...

No

62a. If the previous question is YES: do all surgeons actually work with the protocol used in your hospital? Yes/No

62b. Postoperatively we do not apply (naso)gastric feeding tubes? Yes/No

62c. Patients do not receive bowel preparation (single enema) preoperatively ? Yes/No

62d. Thoracic epidural anesthesia for the surgery is started before and lasts to 2 days post operatively: Yes/No

62e. There are only short-acting anesthesia resources used Yes/No

62f. Post-operatively no opiates are administered Yes/No

62g. Before admission discharge day is planned Yes/No

62h. Is there a complication registration?

Yes, National Surgical Complication Registration (LHCR)

Yes, other registration:………………..

No

62i. Remarks on the above questions? ……………………………………………………………………………………………………………………………………………………………………………………………………………………………………………………………………………………………………………………………………………………………………………………………………………………………………………………………………………………… 62j. If any of the above questions the ‘NO’ field is scored, what is the reason? ……………………………………………………………………………………………………………………….. ……………………………………………………………………………………………………………………….. …………………………………………………………………………………………………………………………

63. How is use of the fast-track protocol promoted and monitored?

Not

By means of availability of a protocol on the ward

By means of the presence of a protocol in the checklist of the medical record

By means of a (digital) database

By means of feedback information from a database

64. How many different departments are involved in the diagnostic phase (s) of the process? For example: Radiology, GE etc?

64a. Number of departments: ..... ... ... ... ... ...

64b. Which departments are involved: ... ... ... ... ... ... ... ... ... ... ... ... ... ... ... ... ... ... ... ... ... ... ... ... ... ... ... ... ... ... ... ... ... ... ... ... ... ... ... ... ... ... ... ... ……………………………………………………….. ………………………………………………………… ……………………………………………………….. ………………………………………………………..

65. How are the data of the patient registered?

In an electronic patient record

There is a mix of an electronic record and a paper file ... ... ... ... ... ... ... ... ... ... ... ... ... ... ... ... ... ... ... ... ... ... ... ... ... ... ... ... ... ... ... ... ... ... ... ... ... ... ... ... ... ... ... ... ... ... ... ... ... ... ... ... ... ... ... ... ... ... ... ... Other: ... ... ... ... ... ... ... ... ... ... ... ... ... ... ... ... ... ... ... ... ... ... ... ... ... ... ... ... ... ... ... ... ... ... ... ... ... ... ... ... ... ... ... ... ... ... ... ...

66. Do you initiate a new patient file for every consultant involved? Yes/No

67. What measures are taken to ensure (exchange of) information for each specialism remains up-to-date? ……………………………………………………………………………………………………………………………………………………………………………………………………………………………………………………………………………………………… ...

**OBSERVATION FORMAT**

Patient satisfaction:

1. Does the hospital/the membership collect data regarding patient satisfaction? Yes/No
2. 2a. Is there an official welcome at the entrance, where an overview of the spaces, the different specialties, departments, and walking routes is presented? Not/limited/average/often/always

2b. Special observations: …………………………………………………………………………………………………………………………………………………………………………………………………………………………………………………………………………………………………………………………

3a. Is there a website with an overview of results concerning quality (e.g. quality indicators and patient ratings)? not/limited/average/often/always

3b. Special observation: ……………………………………………………………………………………………………………………………….

4. Ask an employee what happens to a patient after he/she has completed his/her work and cross options below:

1. The answer is related to work on the department/section of the employee

2. The answer relates to work of employees in other departments

Safety

5a. In a clean and tidy environment materials are easy to find ; various stocks are easy to count or estimate and the work is safer and more efficient. Materials are visibly sorted and the overviews/inventories to quickly find materials are present. Not/limited/average/often/always

5b. Special observations: …………………………………………………………………………………………………………………………………………………………………………………………………………………………………………………………………………………………………………………………………………………………………………………………………………………………………………………………………………………….. 6a. Score the level for lighting, air quality and noise level in the hospital not/limited/average/often/always

6b. Points for improvement: ..............

Visual management system

7. In a properly functioning department elements are present that visual provide cues and instructions. Alerts assist employees performing the right work at the right time at the right place, and help to increase productivity. Internal waiting periods are shortened by, for example, sound or light signals, color encodings, for everyone, accessible instructions and maintenance status reports. Circle the level at which these aspects are applied:

7a. GE Department: not/low/average/often/always

7b. Outpatient surgery: not/low/average/often/always

7c. Radiology: not/low/average/often/always

7d. Operating rooms: not/low/average/often/always

7e. Nursing ward: non/low/average/often/always

7f. Points of improvement: ... ... ... ... ... ... ... ... ... ... ... ... ... ... ... ... ... ... ... ... ... ... ... ... ... ... ... ... ... ... ... ... ... ... ... ... ... ... ... ... ... ... ... ... ... ... ... ... ...

Planning system outpatient clinic and operating theatre

8a. The best plants rely on a single ‘pacing process’ for each product line and its suppliers. This process, usually at the end of the line, controls speed and production for all the upstream activities, much as a pace car sets the speed at a racetrack. Demand for each product at each work center is triggered by demand at the next. This keeps inventory from building up, improves quality, and reduces downtime because production lines aren’t kept waiting for parts.

Circle the level to which this is applied: not/low/average/often/always

8b. Notable: ……………………………………………………………………………………………………………………………………………………………………………………………………………………………………………………………………………………………………………………………………………………………………………………………………………………………………………………………………………………..

Space usage, transport and streamlining

9. In the best organizations all space is efficiently exploited. Materials are moved only once, via the shortest route, using appropriate resources. Materials are only near or on the place where they are used. Instruments and maintenance funds are kept on the place where they should be deployed. Circle the level to which this is applied:

9a. MDL Department: not/low/average/often/always

9b. Outpatient clinic surgery: not/low/average/often/always

9c. Radiology: not/low/average/often/always

9d. Operating rooms: not/low/average/often/always

9e. Nursing ward: non/low/average/often/always

9f. Notable: ……………………………………………………………………………………………………………………………………………………………………………………………………………………………………………………………………………………………………………………………………………………………………………………………………………………………………………………………………………………..

Teamwork and motivation

10. In the best organizations everyone is aware of the organization goals for continuous productivity and quality and everyone knows what is expected of him or her. He or she is also very motivated to help others and to share knowledge. Talk with (a) employee (s) and estimate how someone is involved in his work. Circle:

10a. MDL Department: not/low/average/often/always

10b. Outpatient clinic surgery: not/low/average/often/always

10c. Radiology: not/low/average/often/always

10d. Operating rooms: not/low/average/often/always

10e. Nursing ward: non/low/average/often/always

10f. Notable: ……………………………………………………………………………………………………………………………………………………………………………………………………………………………………………………………………………………………………………………………………………………………………………………………………………………………………………………………………………………..

Condition and management of instruments

11. In the best organizations all instruments clean and well managed. At the acquisition date and the equipment – costs clearly stated (for example with a sticker system) and an overview of the maintenance history is kept for each device. Ask employees how the maintenance and replacement of equipment is regulated. Question or they may say with the purchase of a new instrument. Look at the equipment: old equipment or looking new, says something positive about the maintenance. Circle the level of fitness and management of instruments:

11a. MDL Department: not/low/average/often/always

11b. Outpatient clinic surgery: not/low/average/often/always

11c. Radiology: not/low/average/often/always

11e. Operating rooms: not/low/average/often/always

11f. Nursing ward: non/low/average/often/always

11g. Notable:……………………………..

Management of complexity, variation and acceleration

12. Instructions for assessment are that unnecessary information is collected. When you see that people collect data manually or you see many keyboards for data entry, this might indicate that the complexity is poorly governed. In the best organizations, it is not necessary that employees track the status. Ask employees whether these techniques are applied in their work and circle the level:

12a. MDL Department: not/low/average/often/always

12b. Outpatient clinic surgery: not/low/average/often/always

12c. Radiology: not/low/average/often/always

12d. Operating rooms: not/low/average/often/always

12e. Nursing ward: non/low/average/often/always

12f. Notable: ……………………………………………………………………………………………………………………………………………………………………………………………………………………………………………………………………………………………………………………………………………………… ...
