# Supplementary material for: Development of an instrument to analyze organizational characteristics in multidisciplinary care pathways; the case of colorectal cancer
Source: BMC Res Notes. 2015 Apr 9;8:134. doi: 10.1186/s13104-015-1084-1 (PMC4396572; doi:10.1186/s13104-015-1084-1)
Supplement: Additional file 2: — Sample report. [file 13104_2015_1084_MOESM2_ESM.pdf]

## Illustrative sample of a hospital scoring

### Patient groups

Eight hospitals participated in the survey, of which three were academic, one was a teaching hospital and four were non-teaching hospitals. A number of 472 patients underwent elective surgery due to a primary colorectal carcinoma. All other patients were excluded.

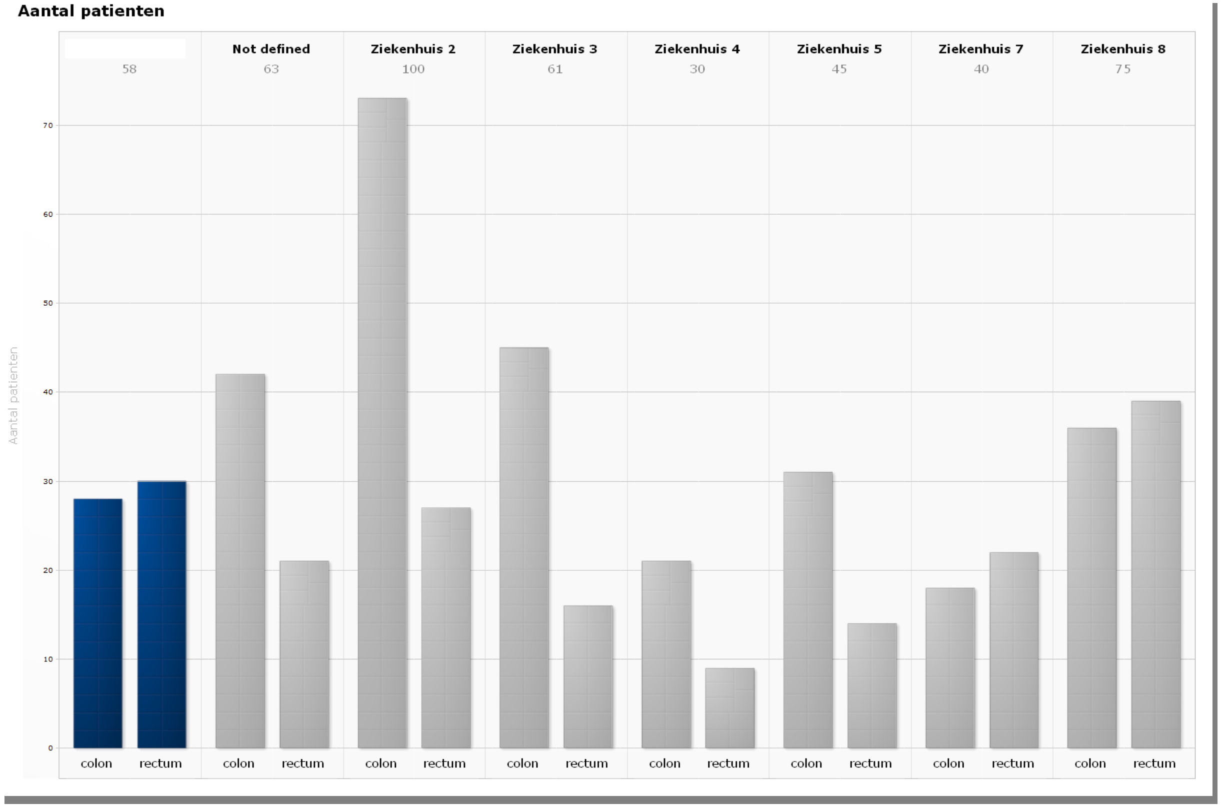


**Table 1.** *Number of included elective patients per hospital with a specification for colon and rectum carcinoma. In the colums the hospital number is presented.'Aantal patiënten' = number of patients.'Ziekenhuis'= hospital.*

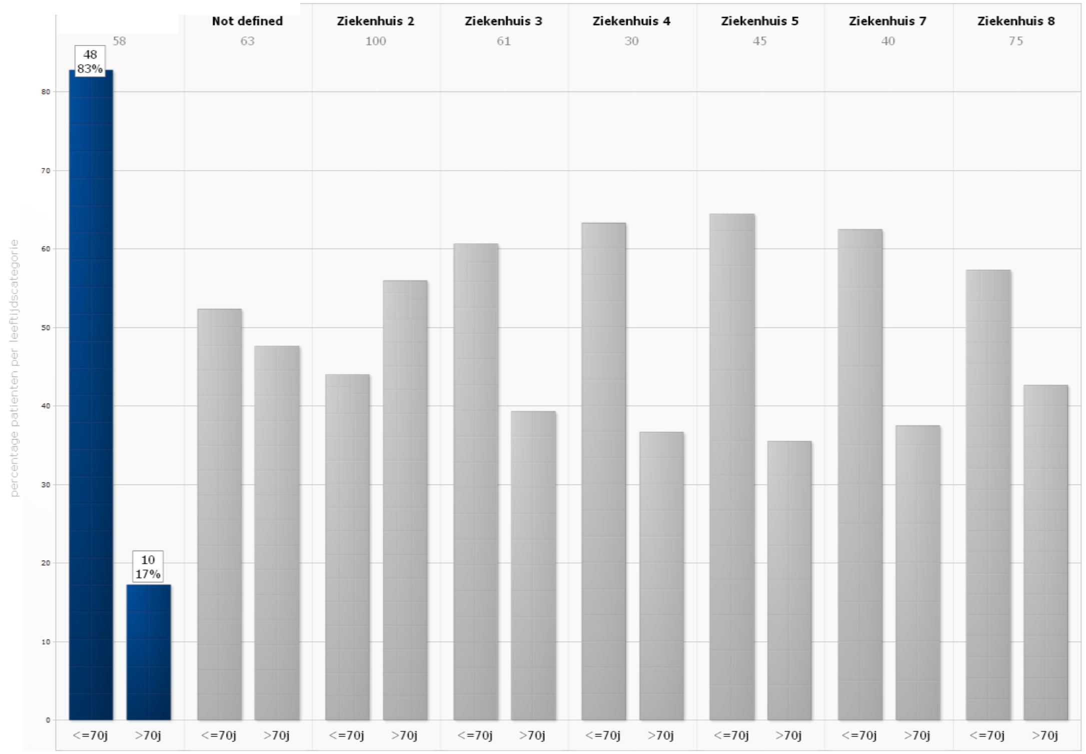


**Table 2.** *Age per hospital during surgery. Divided in < 70 years and > 70 years. In the colums the hospital number is presented. 'Ziekenhuis' = hospital.*

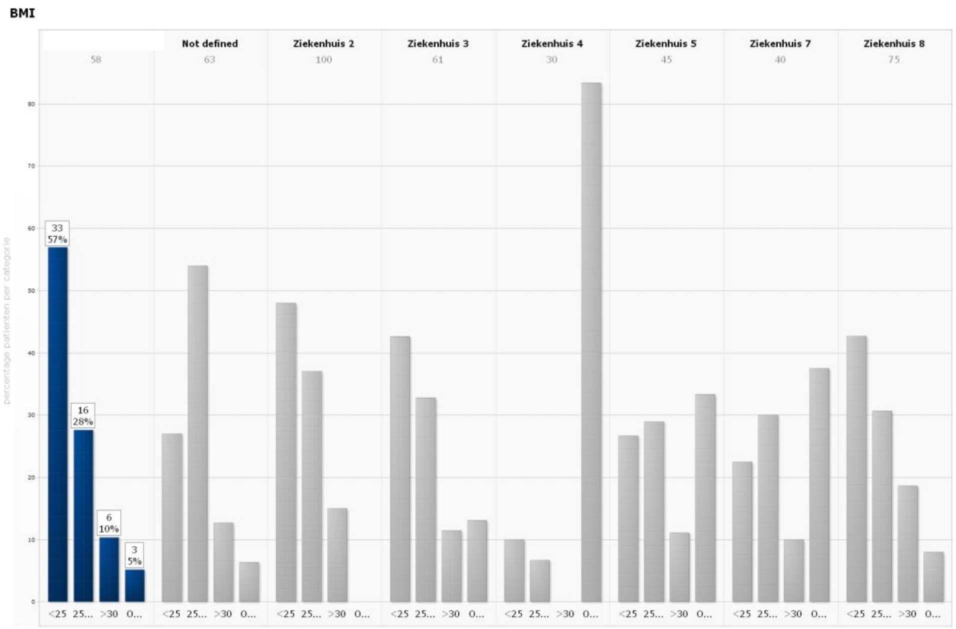


**Table 3.** *Body Mass Index, divided in < 2, between 25 and 30 and > 30.*

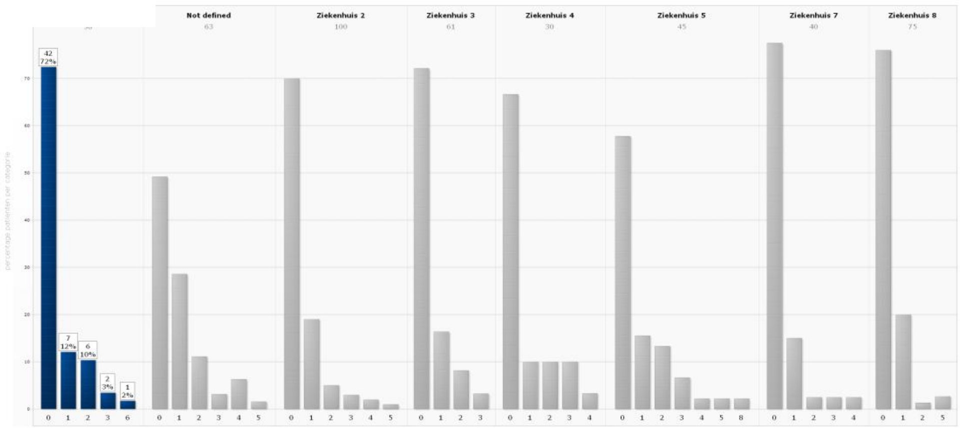


**Table 4.** *Charleston Comorbidity Score.* ‘Ziekenhuis’ = hospital

## Flowchart

A tailor-made flowchart with the patients-tread pathway.

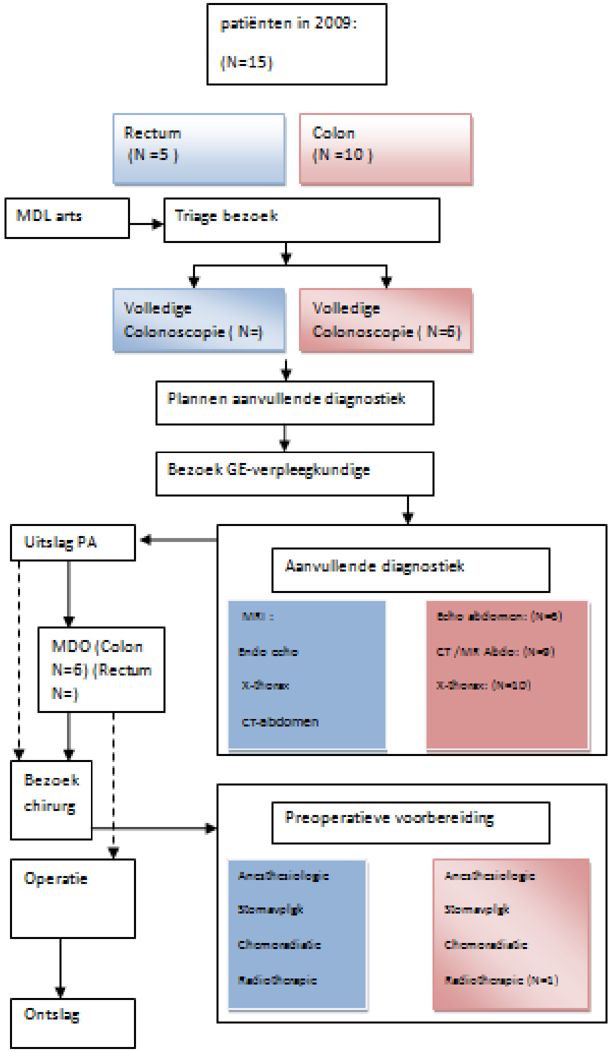


**Table 5.** *Dutch example of a flow chart specified for patients with colon carcinoma and rectum carcinoma in your hospital.*

## Throughput

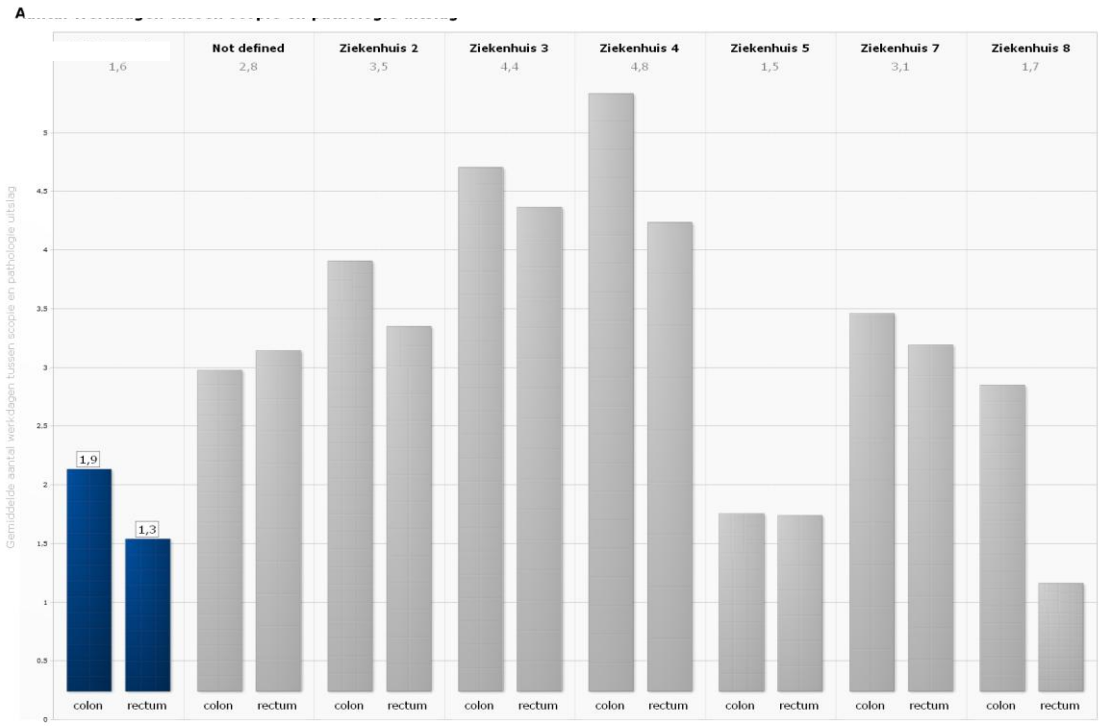


**Table 6.** *Number of workdays between scopy and PA for colon and rectum. 'Ziekenhuis' = hospital.*

## Number of patient visits

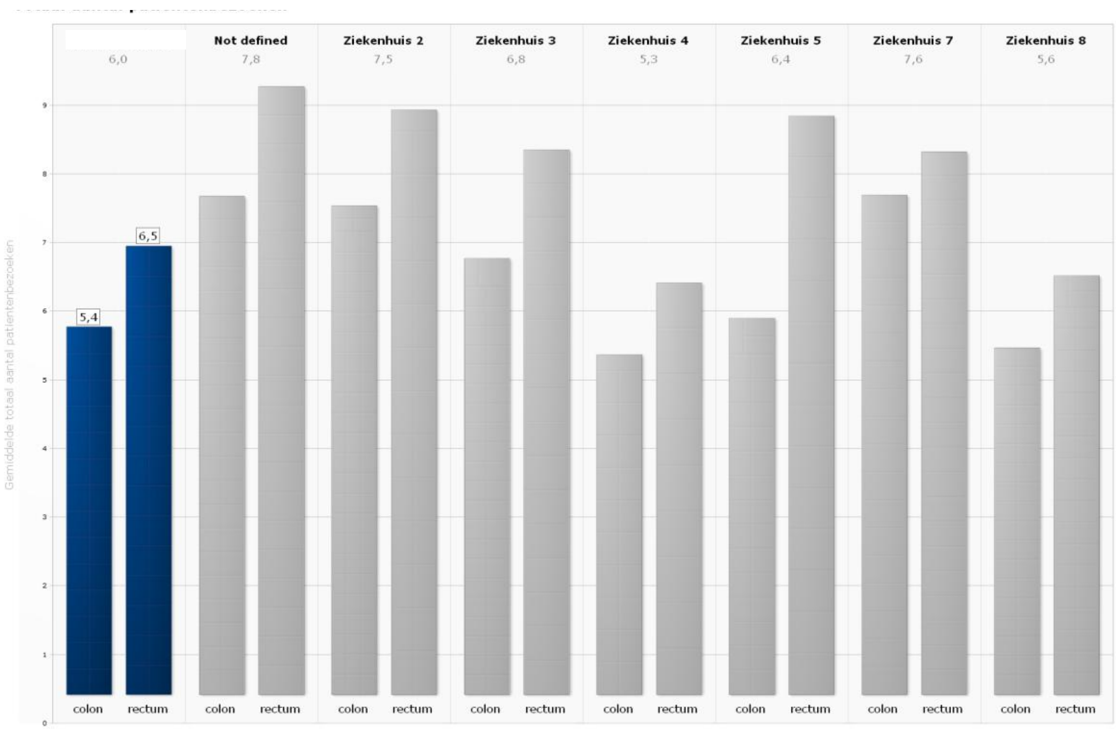


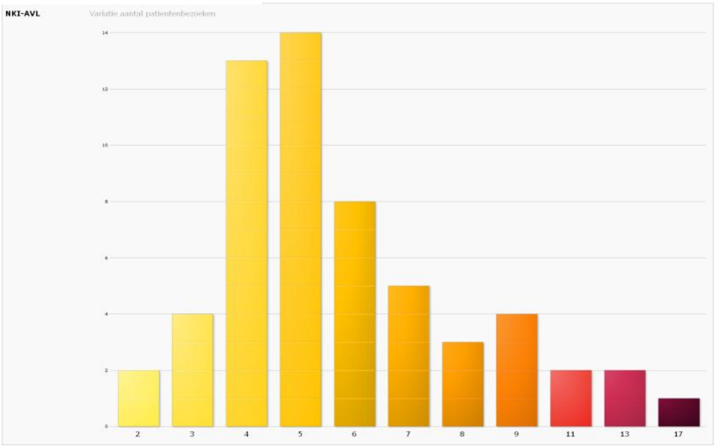


**Table 7 and 8.** *The number of patient visits prior to surgery to outpatient clinics for colon and rectum ( surgery, anesthesiology and GE) and radiology. Variation of the number of visits in your own hospital. 'Ziekenhuis' = hospital.*

## Patient related outcome

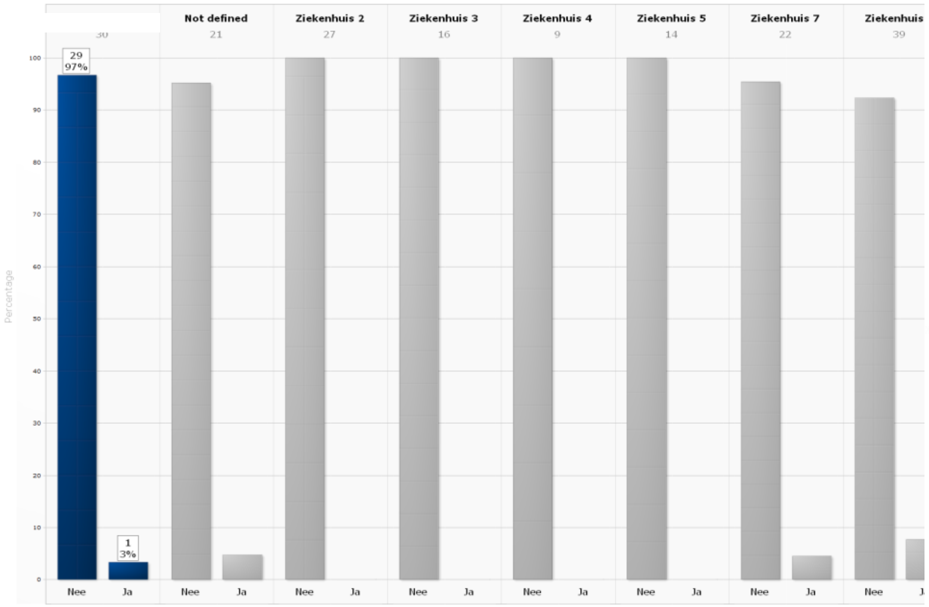


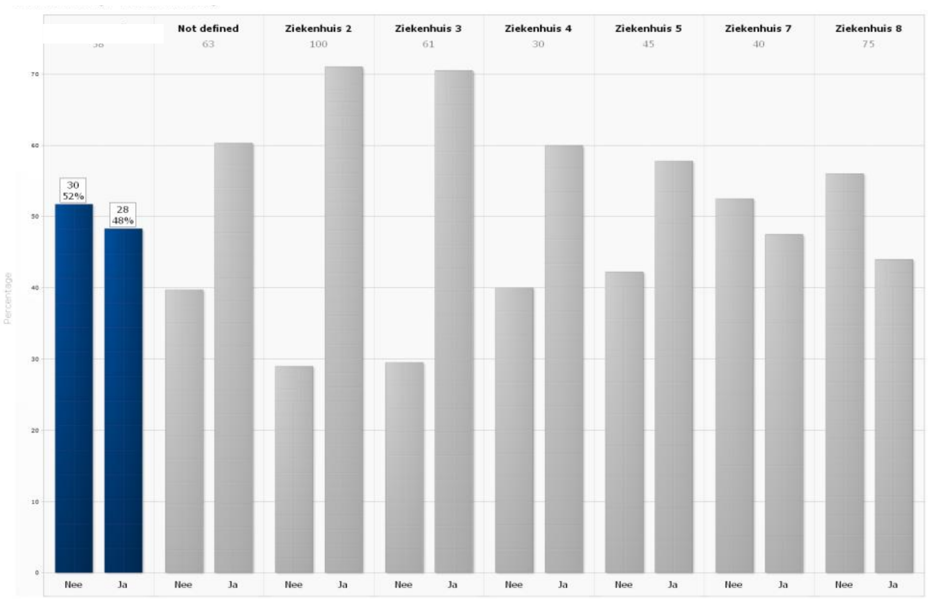


**Table 9 and 10.** *Examples of patient related outcome: pre-operative imaging. liver and lung.Number of patients with imaging ( yes/no). 'Ziekenhuis' = hospital. 'Nee' = no, 'ja' = yes.*

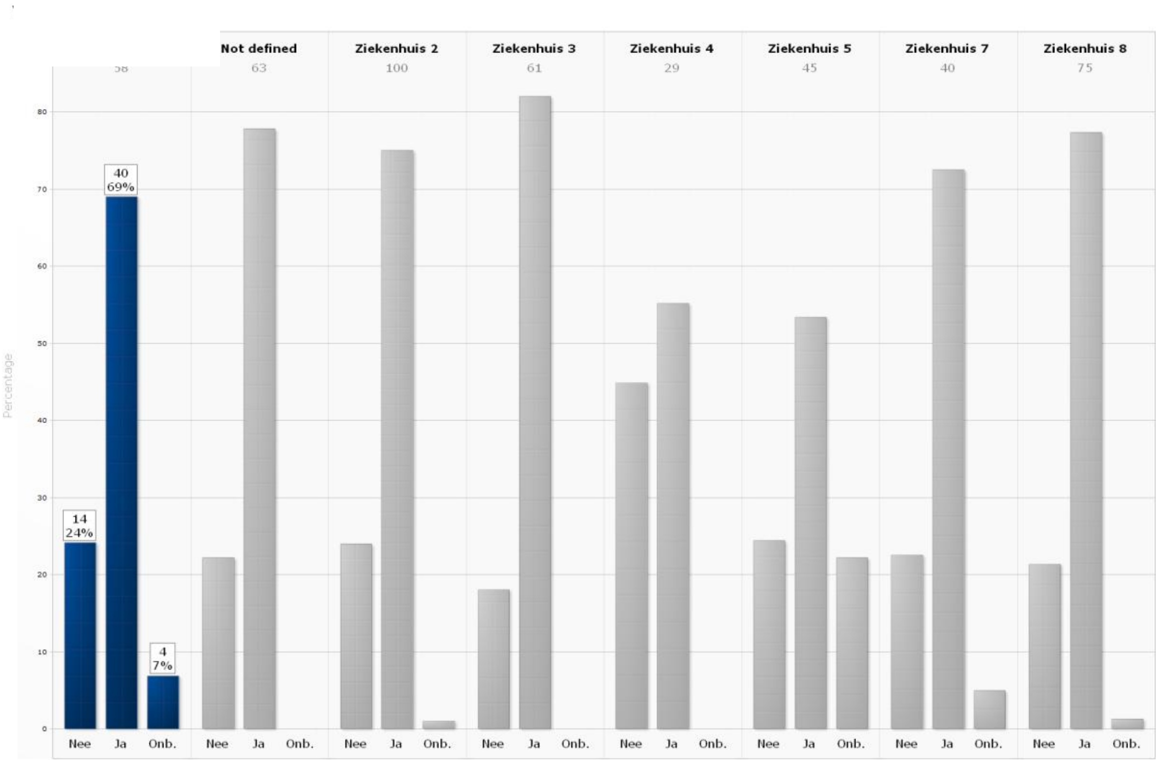


**Table 11.** *Number of patients with a total colosopy ( no/yes/ unknown). 'Ziekenhuis' = hospital, 'Nee' = no, 'ja' = yes.*

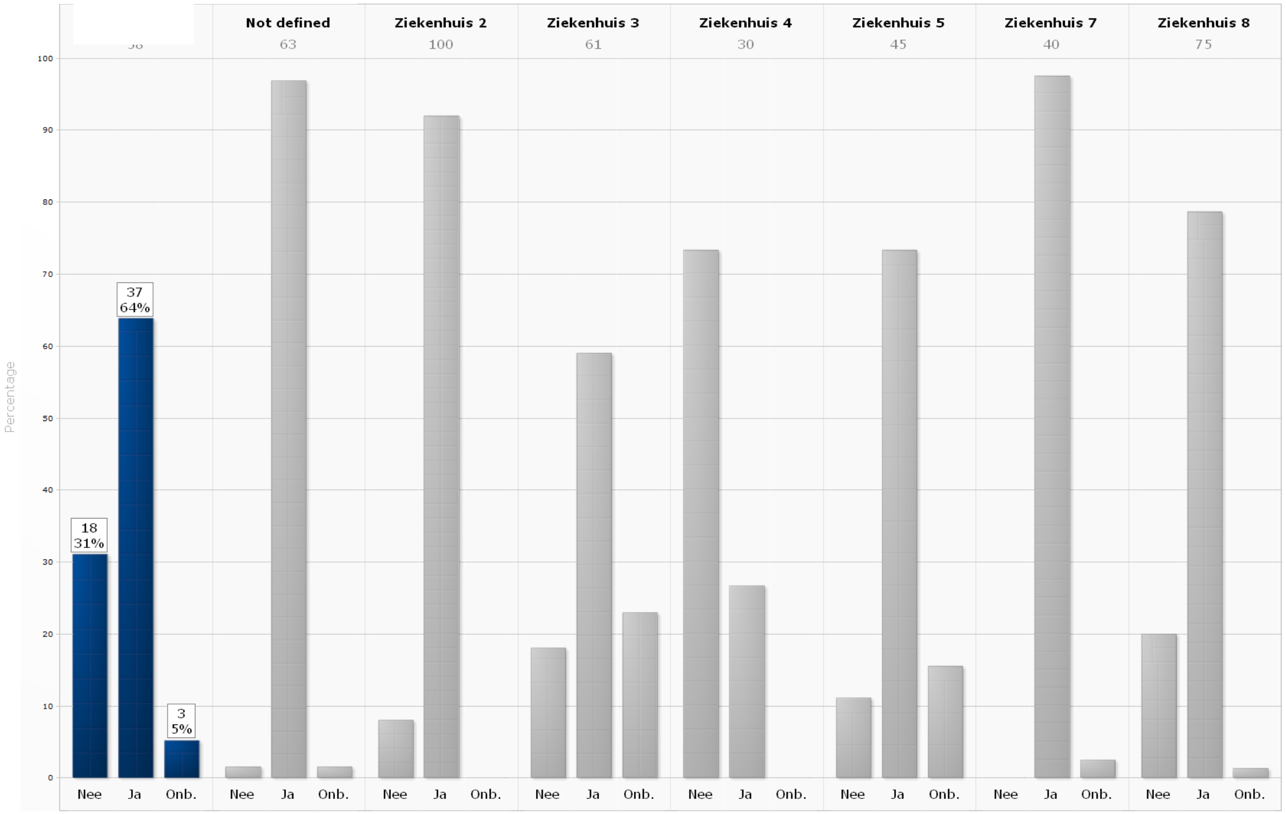


**Table 12.** *Number of patients discussed in MDT ( no/yes/ unknown). 'Ziekenhuis' = hospital. 'Nee' = no, 'Ja' = yes.*

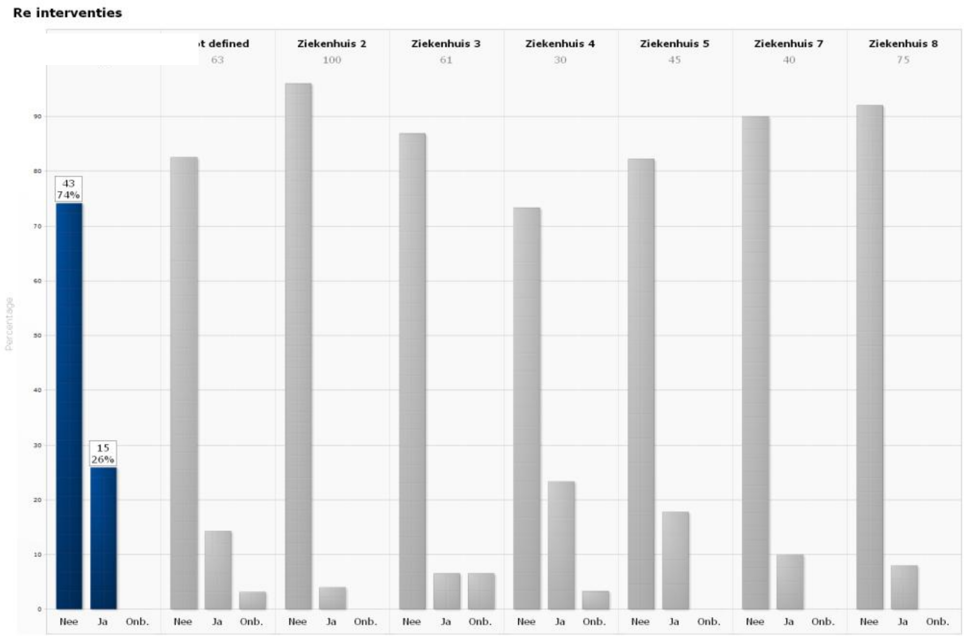


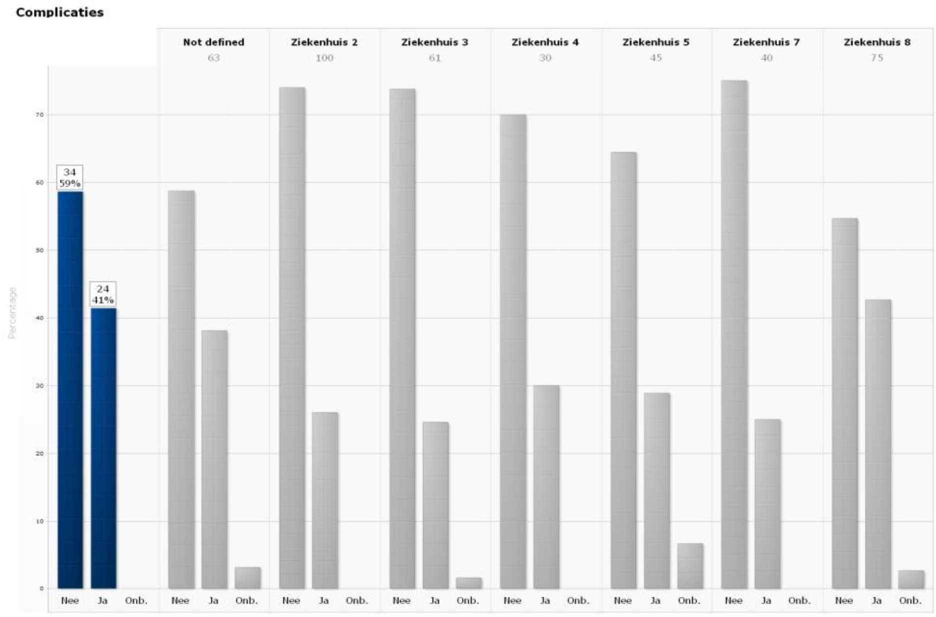


**Table 13 and 14.** *Number of reinterventions and number of complications ( no/yes/ unknown). Ziekenhuis' = hospital. 'Nee' = no, 'Ja' = yes.*

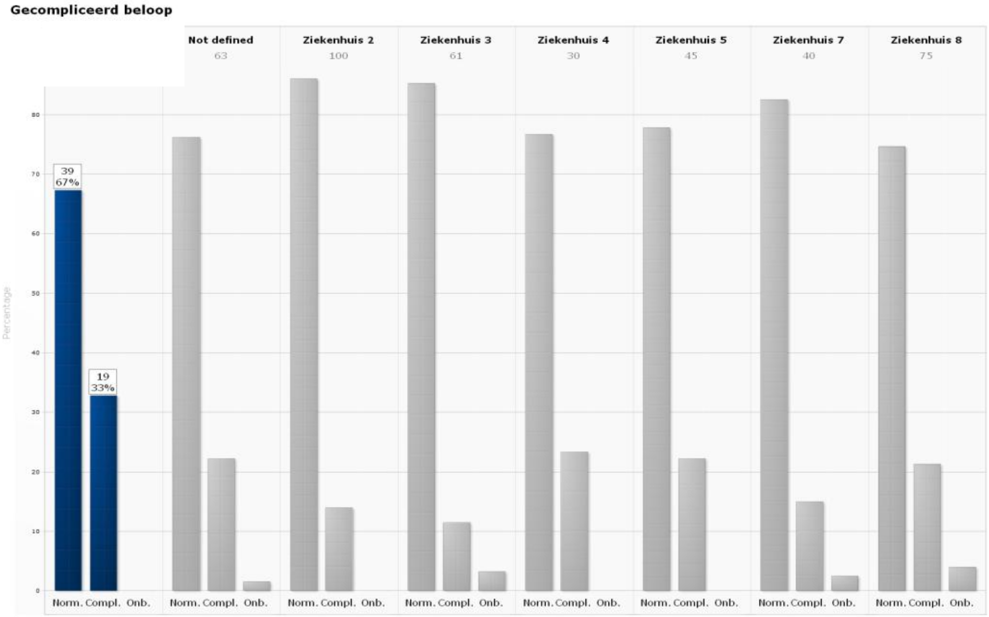


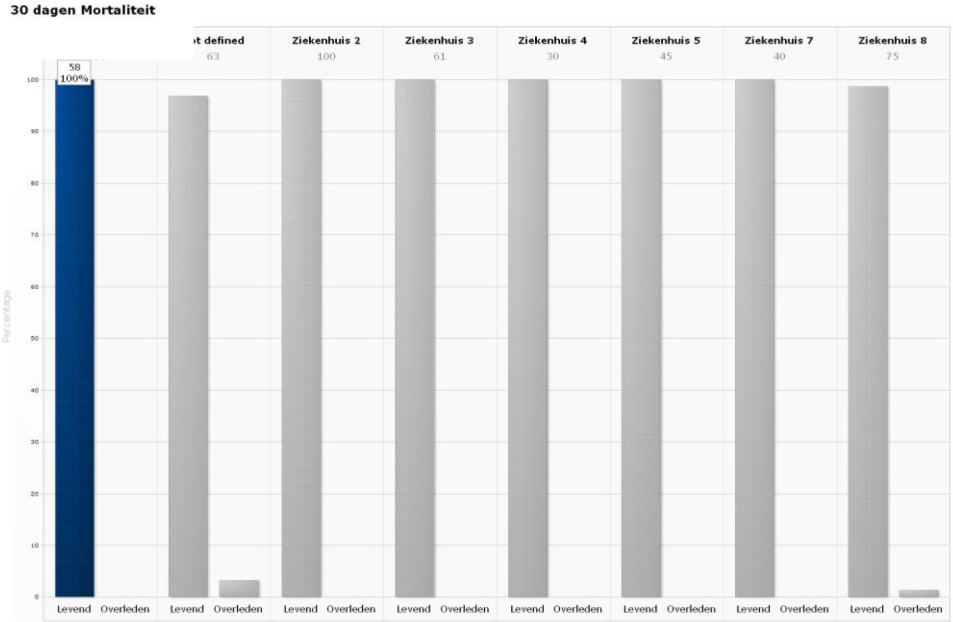


**Table 15 and 16.** *Complicated course and 30-day mortality ( no/yes/unknown).'Gecompliceerd beloop' = complicated course. '30 dagen mortaliteit' = 30-day mortality. 'Ziekenhuis' = hospital.'Levend' = alive. 'Overleden' = deceased.'Ziekenhuis' = hospital.*

Organizational characteristics.

| | Hospital 1 | Hospital 2 | Hospital 3 | Hospital 4 | Hospital 5 | Hospital 6 | Hospital 7 | Hospital 8 | Mean |
|---|---|---|---|---|---|---|---|---|---|
| Operational focus | | | | | | | | | |
| Autonomous work cells | | | | | | | | | |
| Physical lay-out | | | | | | | | | |
| Multi-skilled team members | | | | | | | | | |
| Pull planning | | | | | | | | | |
| Non-value adding activities | | | | | | | | | |

**Table 17.** *Organizational characteristics measured during the in-depth analysis in your hospital.*
